# The effect of external application of palm pollen grains extracts on *phoenix dactylifera* cv. zaghloul fruits quality

**DOI:** 10.1038/s41598-025-14703-8

**Published:** 2025-09-23

**Authors:** Neveen B. Talaat, Mohamed R. A. Nesiem, Ezz G. Gadalla, Shaimaa F. Ali

**Affiliations:** 1 Department of Plant Physiology, Faculty of Agriculture, Cairo University, Giza, Egypt; 2 Central Laboratory for Date Palm Research and Development, Agricultural Research Center, Giza, Egypt

**Keywords:** *Phoenix dactylifera*, Date palm, Biostimulant, Fruit quality, Antioxidant enzymes, Nutrient uptake, Plant sciences, Chemistry

## Abstract

This study was carried out to examine the effect of aqueous or ethanolic date palm pollen grain extract on *Phoenix dactylifera*, cv. Zaghloul fruits. Pollen grain extracts were prepared from male date palm trees cv. Barhi. The experiment was conducted in a completely randomized block design during two consecutive seasons with **three** spraying treatments, i.e. control (distilled water), aqueous or ethanolic date palm pollen grain extract. Each treatment was sprayed twice, i.e. at the Hababouk (fruit’ cell division) and Kimri (fruit’ cell elongation) stages. The results indicated that spraying of 700 ppm aqueous or ethanolic date palm pollen grain extract significantly improved the productivity and quality of ‘Zaghloul’ fruits by increasing the dry matter, crude fiber, ash, total soluble solids, reducing, non-reducing, and total soluble sugars, total carbohydrates, protein, and mineral nutrients concentrations as well as the peroxidase (POD) and catalase (CAT) activities as compared to the control. Also, the external application of aqueous or ethanolic extracts enhanced fruit amino acids acquisition, and total phenols, whereas decreased moisture percentage, titratable acidity, and tannins concentrations in treated date palm fruits. Evidences might indicate that ethanolic date pollen grains extract followed by aqueous extract improve ‘Zaghloul’ fruit yield as compared to control by regulating the nutrients acquisition, sugar accumulation, amino acids profile, and antioxidant response. These findings could support the use of ethanolic or aqueous date pollen grain extract as a bio-stimulant to improve date palm fruit yield and quality.

## Introduction

The date palm (*Phoenix dactylifera* L.) is a perennial tree of great social and economic importance^1^. The fruit of the date palm has nutritional values that are beneficial for consumption and human health. Fruit is considered a food of great importance to the inhabitants of desert areas. While, suitable areas are located within a geographic belt between 24 and 34° N^2,3^. The fruits of date palm are the most widely consumed fruit in the world, especially in the Middle East, North Africa, Asia, and America as it is considered a good source of simple carbohydrates, essential minerals, protein, lipid, phenolic and antioxidant compounds; however, the amounts of these components vary among cultivars and depend on the maturity stage^4,5^.

Date palm pollen grains (DPPGs) are a fine powder and each pollen grain composed of water (5–36%) and solids (64–95%). It has interesting nutritional value, including vitamins, minerals, sugars, lipids, growth factors, enzymes, and co-factors^6^. DPPGs are rich in nutritional supplements due to their content of fatty acids and flavonoids that play an important role as antioxidants^7^. Moreover, DPPGs have a history of use in traditional herbal medicine, and have been reported to exhibit a broad spectrum of antimicrobial properties^8^.

Even today, very few studies have looked at date palm pollen extract as bio-stimulant for improving date palm productivity. Therefore, the current study was conducted to assess the impact of applying aqueous or ethanol date pollen extract, as spraying treatment on physical (dry matter, crude fiber, ash, moisture, total soluble solids and titratable acidity), and biochemical characteristics including reducing, non-reducing, and total sugar, carbohydrate, as well as protein concentrations, amino acid profile, mineral nutrients, total phenols and tannins concentrations as well as peroxidase and catalase activity of treated Zaghloul fruits. Accordingly, it has been hypothesized that the use of aqueous or ethanolic palm pollen grain extract could improve ‘Zaghloul’ fruit yield and quality.

## Materials and methods

### Plant material and experimental procedures

This study was carried out on 9 female date palm trees cv. ‘Zaghloul’ (*Phoenix dactylifera* L.); 15 years old growing in clay soil through two seasons 2020 and 2021 in the experimental orchard of the Agriculture Research Center, Ministry of Agriculture, Giza, Egypt. On the other hand, date palm pollen grains cv. (Barhi) was obtained from East Owainat in the south of the valley in Western Egypt. The experiment included three treatments as follows: (i) control (distilled water); (ii) pollen grain aqueous (PGA) extract; and (iii) pollen grain ethanolic (PGE) extract. **Characteristics of date pollen grains**:

### Pollen grains viability

#### a. Acetocarmine

Determination of date pollen grains viability by using 2% solution of acetocarmine (Sigma-Aldrich, Poznań, Poland) comprising 2 g of carmine in a mixture of 45 ML glacial acetic acid and 55 ML distilled water. Date pollen grains viability was examined by using two drops of a 2% solution of acetocarmine, under a microscope at 200X magnification power. Pollen grains stained red were considered viable, whereas the colorless pollen grains were considered non-viable according to Skrzypkowski et al. (2023)^9^. The pollen viability percentage was.

calculated by the following formula.

$$Pollen \; grain \; viability \; \% =\frac{Viable \; pollen}{Total \; pollen} \times 100$$

#### B. Pollen grains germination

The medium of pollen grain germination contained 10% sucrose, 150 ppm boric acid, and 2.5 g/l gelrite in petri dishes, pH 7. The pollen grains were cultured on medium in an incubator at 25 °C for 24 h. Pollen germination was examined under a microscope, where appearance of pollen tubes classified as germinated according to Ismail (2014) and Skrzypkowski et al. (2023)^9,10^. Pollens germination percentage was calculated by the following equation.

$$Pollen \; grain \; germination \; \% \; = \frac{Numder\;of\;germinated\;pollen\;grains\;per\;field}{Total \; number\;of\;pollen\;grains\;per\;field} \; \times 100$$

Pollen grain germination % =.

##### Palm pollen grains extract

Male bunches (cv. Barhi) were collected at maturity during March and then moved to shaded area for good air drying, through separating spathes from bunches and spreading on clean papers. Spathes were frequently changed from one paper to another to get rid of moisture. After good drying, pollen grains were collected for testing.

- **Aqueous extract**: A 7 g of palm pollen grain was well mixed with 100 ML of distilled water and set aside for 72 h under stirring by an orbital shaker. After that, the mixture was twice filtered by using filter paper Whatman No. 1, and non-absorbent cotton. The filtrate was completed again to 100 ML by distilled water and was used as stock solution. The stock solution was immediately used or stored at ‒20 °C until use. The desired concentration (700 ppm) was obtained by taken 10 ML from stock solution and completed to 1 L by distilled water^11^.
- **Ethanolic extract**: A 7 g of palm pollen grain was well mixed with 100 ML of ethanol 70% and set aside for 72 h under stirring with an orbital shaker. The solution was twice filtered by using filter paper Whatman No.1, and non-absorbent cotton. The filtrate was evaporated with a vacuum rotary evaporator at 30 °C. The obtained aqueous extract was completed to 100 ML by distilled water (stock solution). The stock solution was immediately used or stored at ‒20 °C until use. The desired concentration (700 ppm) was obtained by taken 10 ML from stock solution and completed to 1 L by distilled water^11^.

The different chemical constituents of aqueous or ethanolic Barhi palm pollen grain extract were determined as follow:

##### Total protein

Total protein was determined according to the method of Lowry et al. (1951)^12^. The blue color was developed by using a Folin phenol reagent, and the absorbance was read at 660 nm, using a spectrophotometer (Shimadzu UV-Visible 1800, Kyoto, Japan).

##### Antioxidant activity

The activity of diphenyl-1-picrylhydrazyl (DPPH) free radical scavenging was determined according the modified method of Barros et al. (2007)^13^. About 1 ML of DPPH (0.1 mM) was dissolved in methanol then added to a 0.5 ML of sample extract at different concentrations. Samples were kept at room temperature for 30 min in the dark and by using a spectrophotometer at 517 nm, the decrease in absorption was measured against blank (DPPH solution without sample).

##### Mineral nutrients

Both pollen grains extracts were digested with H_2_SO_4_ and H_2_O_2_. Nitrogen concentration was determined using the micro-Kjeldahl method, as described by Wang et al. (2016)^14^. Using a spectrophotometer (Shimadzu UV-Visible 1800, Kyoto, Japan) at a wavelength of 405 nm, the phosphorus concentration was determined using the vanadomolybdate method, as described by Wieczorek et al. (2022)^15^. A flame photometer (SKZ International Co., Ltd., Jinan Shandong, China) was used to determine the potassium and sodium^16^. According to Stafilov and Karadjova (2009)^17^, calcium at 422.8 nm, magnesium at 285.2 nm, iron at 248.3 nm, zinc at 213.9 nm, and manganese at 279.5 nm were determined on an atomic absorption spectrometer, iCE 3300 AA of Thermo Scientific.

##### Amino acid analysis

Various amino acids (glutamic acid, aspartic acid, proline, glycine, alanine, arginine, cysteine, histidine, isoleucine, leucine, lysine, methionine, phenylalanine, serine, threonine, tyrosine, and valine) were determined according to the method of Laurey (1997)^18^. Both sample extracts and standard were hydrolyzed by vapors of HCl for 20 h at a temperature of 110 °C. After hydrolysis, samples were extracted three times with 100 µl of 40% acetonitrile and 0.5% trifluoroacetic acid. The extracts were completely dried in a Speed Vac and re-dissolved in a sample buffer. The samples and standards were analyzed using the amino acid analyzer (Beckman 6300 system, New York, Valhalla, NY).

### Experimental design

The experiment was designed as a completely randomized design with three replicates. The experiment included three treatments as follows: (i) control (distilled water); (ii) pollen grain aqueous extract (PGA) (700 ppm); and (iii) pollen grain ethanol extract (PGE) (700 ppm). Each studied extract was sprayed at early morning. To make sure optimal penetration into fruit tissues, 0.1% (v/v) tween-20 was added as a surfactant. The ´Zaghloul´ trees were in vigor, healthy, good physical condition, and free of insects, damage, or diseases. All the selected date palms received the same common horticultural practices that are usually applied in the orchard. Each spraying treatment was done twice, at the Hababouk stage (the first stage of fruit development; April and May) and the Kimri stage (beginning of the fruit mature stage; May–July).

### Plant productivity analysis

After 177 days from pollination, bunches of palm fruits were harvested and weighed. A sample of 60 fruits for each replicate at the Tamar stage was randomly selected, washed, then dried, and weighed. Furthermore, fruit volume (cm^3^) through a graduated cylinder and dimensions (length and diameter, cm) by a vernier caliper were measured. The fruits and seeds, flesh weight was separated and weighed (g) as well as fruit flesh pulp (%) was calculated. Fruit flesh weight was calculated as fruit weight minus seed weight. In addition, the seed/fruit ratio was calculated. The following physical and chemical characteristics for fruits from each treatment were determined:

#### Moisture, dry matter, crude fiber and Ash determination

Determination of moisture, dry matter, crude fiber, and ash concentrations were done according to the official methods as described by the Association of Official Analytical Chemistry^19^.

#### Total soluble solids (TSS) and titratable acidity (TA) determination

Total soluble solids (TSS %) of the fruit juice was determined by a digital refractometer (DR 6000, A. Kruss Optronic GmbH, Hamburg, Germany). Titratable acidity (TA %) was measured in fruit juice as a percentage by titration with 0.1 NaOH in the presence of phenolphthalein as an indicator and the results were converted to malic acid % as the dominant organic acid in the fruit^20^.

#### Reducing, non-reducing, and total sugars determination

A 100 ML of distilled water was used to homogenize 3 g of fresh date pulp. The homogenate was centrifuged for 5 min. at 3000 rpm and the supernatant was used. The phenol-sulfuric acid method was used to measure the amount of total sugar^21^. The dinitrosalicylic acid method was used to determine the reducing sugar^22^. Non-reducing sugar was calculated as the difference between total and reducing sugars. Different sugar concentrations were determined as mg glucose/g date fruit fresh weight.

#### Determination of total carbohydrate and total protein

Total carbohydrates were extracted and determined according to the methods of Yih and Clark (1965) and DuBois et al. (1956)^21,23^, respectively. On the other hand, total protein concentrations were measured according to the method of Lowry et al. (1951)^12^.

#### Amino acid analysis

Date palm fruit samples and standard were hydrolyzed by vapors of HCl for 20 h at a temperature of 110 °C. After hydrolysis, samples were extracted three times with 100 µl of 40% acetonitrile and 0.5% trifluoroacetic acid. Next, the extracts were completely dried in a Speed Vac and re-dissolved in a sample buffer. The samples and standards were analyzed using the amino acid analyzer (Beckman 6300 system, New York, Valhalla, NY) according to the method of Laurey (1997)^18^.

#### Total phenols and tannins determinations

The total phenols concentration was determined according to the Folin–Ciocalteu assay as described by Singleton et al. (1999)^24^. The standard curve for total phenols was prepared using gallic acid. The total phenols were expressed as mg gallic acid equivalents (mg gallic acid/100 g fresh weight). On the other hand, the colorimetric technique described by Bentebba et al. (2020)^25^ was used to determine the concentrations of tannins in the date fruit extracts. Total tannins concentration was expressed in mg of catechin equivalent (mg catechin/100 g fresh weight).

#### Determination of catalase (EC 1.1.1.6) and peroxidase (EC 1.11.1.7) activities

Fresh date pulp (2 g) was homogenized with 5 ML of ice-cold 100 mM phosphate buffer (pH 7.4) containing 1% polyvinyl pyrrolidine and 1 mM EDTA. The homogenate was centrifuged at 15,000 rpm for 10 min at 25 °C. The supernatant was collected and used to measure catalase (CAT; EC 1.11.1.6) and peroxidase (POD; EC 1.11.1.7) activities. Monitoring the decrease in absorbance at 240 nm resulting from H_2_O_2_ decomposition was used to measure CAT activity^26^. By analyzing the oxidation of guaiacol at 470 nm, the peroxidase activity was determined as described by Hemeda and Klein (1990)^27^.

#### Mineral nutrients determination

Fresh fruit pulp (3 g) was dried in an oven at 70 °C until its weight was consistent and crushed. Samples were digested with H_2_SO4 and H_2_O_2_. Nitrogen, phosphorus, potassium, sodium, calcium, magnesium, iron, zinc and manganese concentrations were determined as mentioned above with pollen grains extract.

### Statistical analysis

One-way analysis of variance (ANOVA) was used to analyze the obtained data. Data were analyzed based on a randomized complete block design with three replications. Since the data of the two seasons had similar trend, a joint analysis was conducted for both. Duncan’s multiple range test was used to determine the statistical significance of the means at *p* < 0.05 as described by Duncan (1955)^28^. Data were presented as means ± standard error (SE).

### Statement on guidelines

The used plant materials in this research were conducted following the relevant guidelines and regulations. Approvals and permissions were obtained from farm owners before samples collection under the supervision of the Central Laboratory for Date Palm Researches and Development – Agricultural Research Center, Giza, Egypt.

### Data Availability

The authors declare that the raw data supporting the findings of this study are available and can be requested from the corresponding author when needed.”

## Results

### Pollen grains viability

**Fig. 1:**
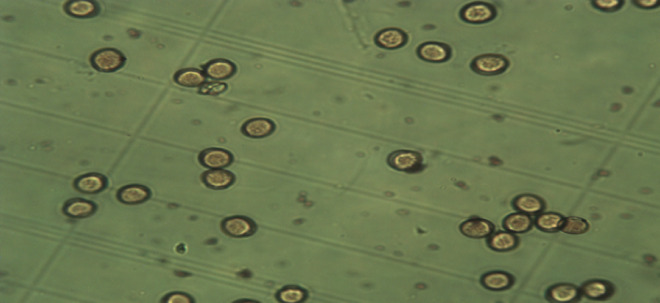
Date palm pollen grain cv. Barhi viability percentage by acetocarmine method.

**Fig. 2:**
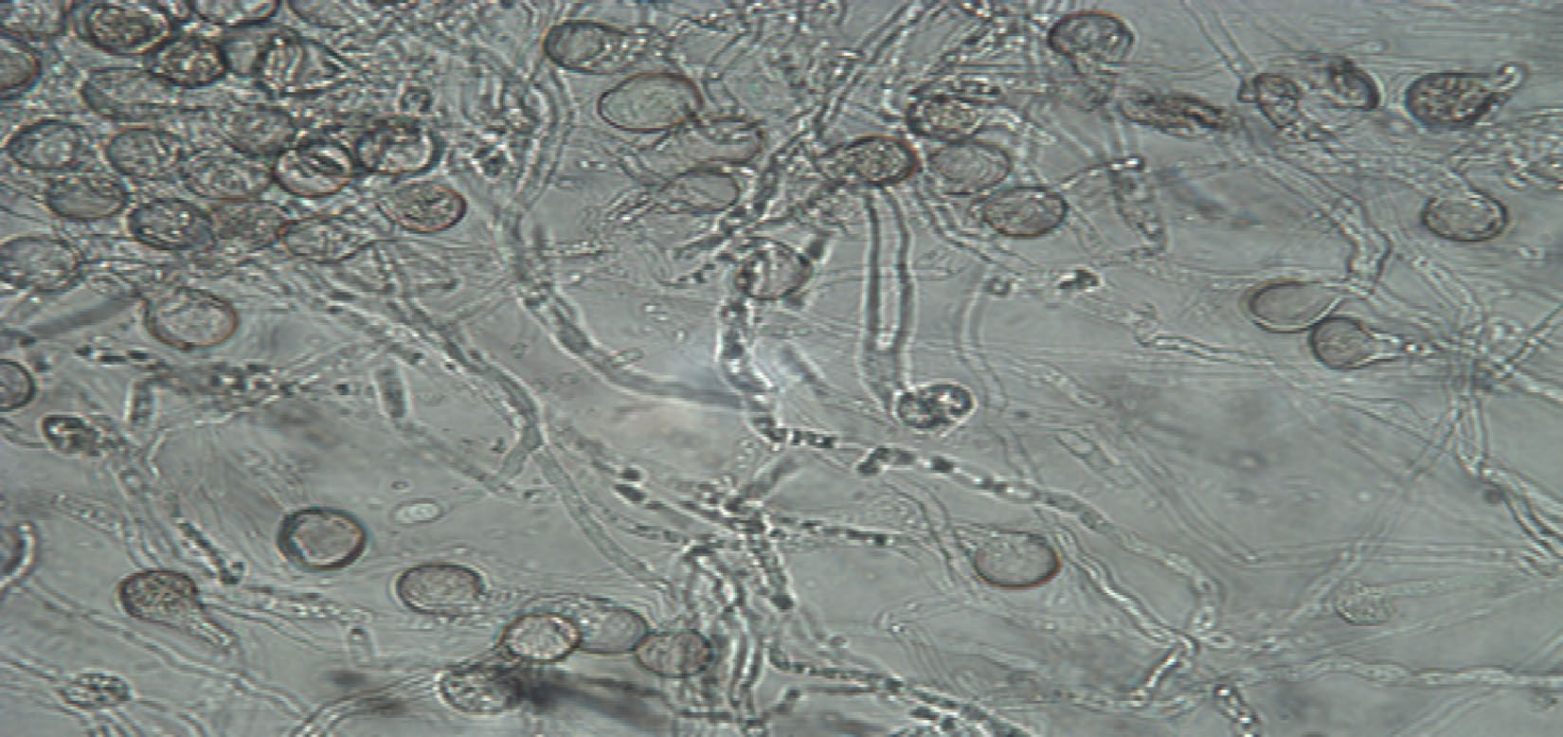
Date palm pollen grain cv. Barhi viability percentage by germination method.

The viability percentage of date palm pollen grain cv. Barhi by acetocarmine and germination reached about 96% as shown in Figs. (1 and 2). The tested pollen grains were used for preparation of either aqueous or ethanolic extract.

### Chemical characteristics of pollen grains extract

The chemical composition of date palm pollen grains aqueous (PGA) extract and date palm pollen grain ethanolic (PGE) extract are presented in Table 1.

**Table 1:** Chemical composition of aqueous and ethanol extracts of Barhi date palm pollen grains (on a dry weight basis).

Constituent	Aqueous	Ethanol	Constituent	Aqueous	Ethanol
**1- Protein (mg/g dry weight) DW()**	20.78	29.78	**5- Amino acids (mg/g dry weight) DW)(**		
**2- Antioxidant (DPPH) (mg/ML)**	0.20	0.60	Isoleucine	11.32	15.16
**3- Carbon (%)**	18.00	26.66	Leucine	15.71	24.23
**4- Mineral nutrients (mg/g dry weight) DW)(**			Lysine	14.97	24.56
Nitrogen	27.15	48.03	Phenylalanine	9.27	11.94
Phosphorus	0.46	0.60	Threonine	11.90	13.20
Potassium	3.35	5.43	Valine	12.67	18.90
Calcium	4.84	6.55	Histidine	7.73	10.29
Magnesium	0.07	0.28	Methionine	2.95	4.91
Sulfur	0.32	0.45	Alanine	18.57	32.25
Sodium	0.16	0.16	Arginine	8.88	10.74
Boron	0.19	0.30	Aspartic acid	20.80	32.13
Zinc	0.11	0.20	Glutamic acid	19.39	29.16
Iron	0.10	0.20	Glycine	41.51	55.36
Manganese	0.19	0.27	Serine	10.41	13.93
			Cystine	2.39	4.06
			Tyrosine	0.27	0.45
			Proline	9.65	15.34

### Date fruit physical characteristics

**Fig. 3:**
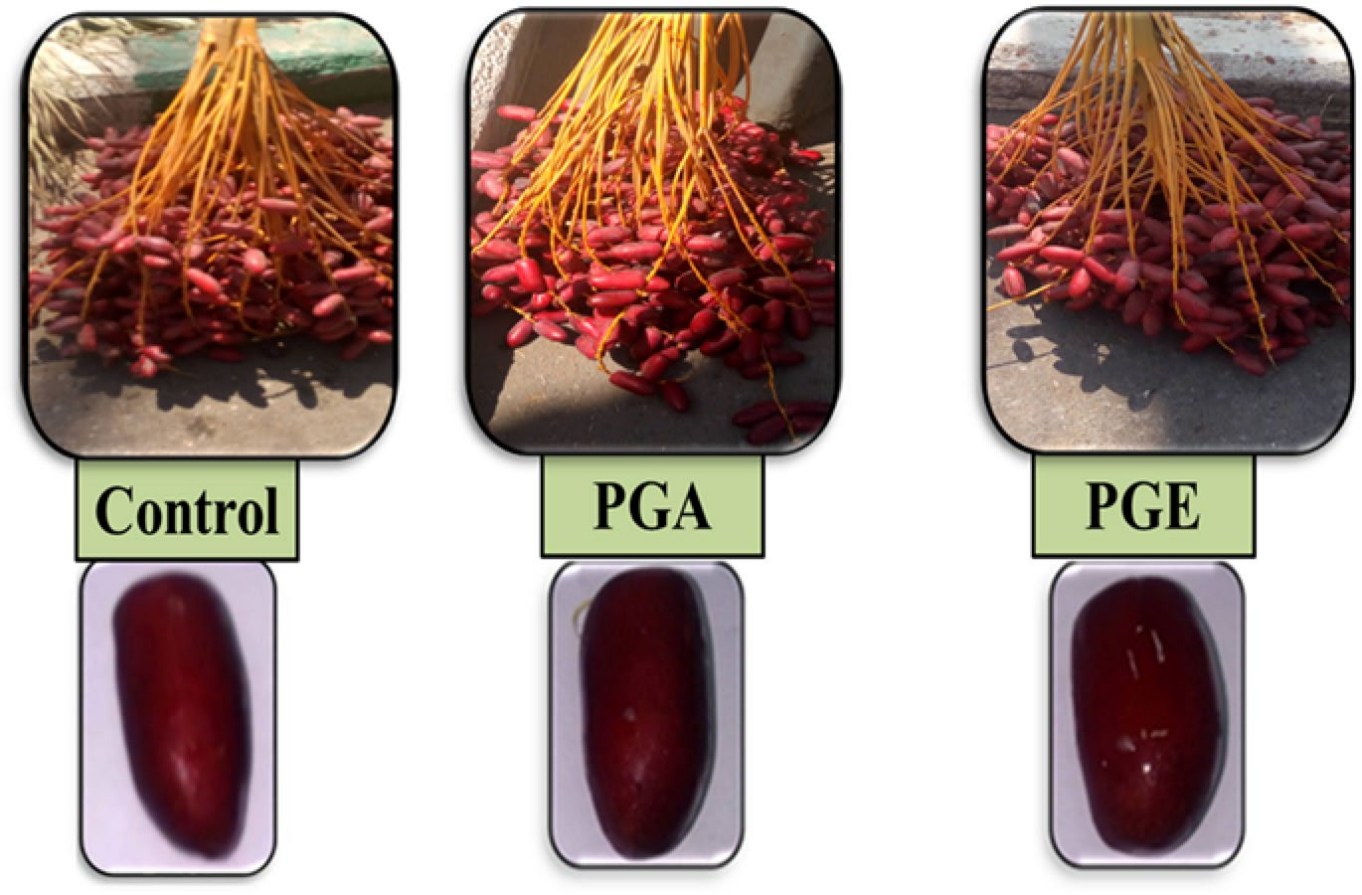
Growth and production of Zaghloul date palm fruits as affected by exogenous spray of pollen grain aqueous **(PGA)** or ethanolic **(PGE)** extract on fruits during Hababouk and Kimiri fruit development stages.

The obtained data indicated that spraying pollen grain extracts (aqueous or ethanolic) significantly increased the fruit growth characteristics of ‘Zaghloul’ dates more than the control treatment (Table 2; Fig. 3). A significant increase in values of ethanolic extract treatment over control were obtained in the bunch weight, fruit volume, fruit length, fruit diameter, flesh weight, and flesh percentage reaching 38, 31, 6, 19, 2 and 5% whereas the increase of aqueous extract treatment over control reaching 15, 9, 4, 10, 4 and 4%, respectively. On the other hand, the date fruits treated with pollen extracts showed lower values of the seed weight, and seed weight/fruit weight ration reaching over control 32 and 30% by ethanolic pollen extract and 32 and 24% by aqueous pollen extract, respectively.

**Table 2:** The bunch weight **(kg)**, fruit weight **(g)**, fruit volume **(cm**^**3**^**)**, fruit length **(cm)**, fruit diameter **(cm)**, flesh weight **(g)**, flesh **(%)**, seed weight **(g)**, and seed weight/fruit weight ratio of ‘zaghloul’ date palm fruits as affected by exogenous applications of pollen grain of aqueous **(PGA)** or ethanolic **(PGE)** extract.

Exogenous application treatment	Bunch weight (kg)	Fruit weight (g)	Fruit volume (cm3)	Fruit length (cm)	Fruit diameter (cm)	Flesh weight (g)	Flesh (%)	Seed weight (g)	Seed weight /Fruit weight ratio
**Control**	4.70 ± 0.1^c^	15.10 ± 0.03^a^	12.83 ± 1.3^b^	4.91 ± 0.1^b^	2.11 ± 0.1^b^	12.86 ± 0.1^a^	85.40 ± 0.7^b^	2.17 ± 0.1^a^	14.60 ± 0.7^a^
**PGA**	5.41 ± 0.1^b^	13.87 ± 0.2^b^	14.66 ± 0.3^ab^	5.08 ± 0.0^ab^	2.29 ± 0.1^ab^	12.35 ± 0.1^a^	89.04 ± 0.8^a^	1.47 ± 0.1^b^	12.40 ± 0.1^a^
**PGE**	6.45 ± 0.4^a^	14.64 ± 0.4^ab^	17.66 ± 1.2^a^	5.24 ± 0.1^a^	2.45 ± 0.1^a^	13.16 ± 0.5^a^	89.89 ± 0.8^a^	1.52 ± 0.1^b^	13.20 ± 0.5^a^

Values are mean ± standard error (*n* = 3). Letters within the same column are statistically different at *p* < 0.05 level following the Duncan’s test.

### Date fruit chemical characteristics

#### Date fruit chemical properties

The externally applied aqueous or ethanolic treatment led to a significant increase in chemical properties of the fruits as compared to the control (Table 3). Application of aqueous or ethanolic pollen extract significantly increased the dry matter, crude fiber, and ash, as well as total soluble solids (TSS) percentage. The increase percentage over control reached 35, 80, 77, and 21% by ethanolic extract treatment and 14, 20, 53, and 15% by aqueous extract treatment, respectively. Meanwhile, aqueous or ethanolic treatment lowered the moisture and titratable acidity (TA) percentage. The lowering reduction reached 15 and 50% by ethanolic extract treatment and 6 and 50% by aqueous extract treatment, respectively, as compared to the control treatment.

**Table 3:** The percentage of moisture, dry matter, crude fiber, ash, total soluble solids **(TSS)**, and titratable acidity **(TA)** as well as total soluble solids/titratable acidity **(TSS/TA)** ratio of ‘zaghloul’ date palm fruits as affected by exogenous applications of pollen grain of aqueous **(PGA)** or ethanolic **(PGE)** extract.

Exogenous application treatment	Moisture (%)	Dry matter (%)	Crude fiber (%)	Ash (%)	Total Soluble Solid (TSS) (%)	Titratable acidity (TA) (%)	TSS/TA Ratio
**Control**	70.50 ± 0.2^a^	29.50 ± 0.2^c^	0.47 ± 0.01^c^	2.65 ± 0.06^b^	26.62 ± 0.3^b^	0.470 ± 0.01^a^	55.30 ± 0.5^b^
**PGA**	66.50 ± 0.2^b^	33.50 ± 0.2^b^	0.63 ± 0.01^b^	4.64 ± 0.04^a^	30.64 ± 0.8^a^	0.21 ± 0.01^b^	153.00 ± 0.8^a^
**PGE**	60.10 ± 0.3^c^	39.70 ± 0.3^a^	0.86 ± 0.03^a^	5.30 ± 0.04^a^	32.33 ± 0.6^a^	0.18 ± 0.01^b^	161.50 ± 0.9^a^

Values are mean ± standard error (*n* = 3). Letters within the same column are statistically different at *p* < 0.05 level following the Duncan’s test.

#### Reducing, non-reducing and total sugar concentrations

**Fig. 4:**
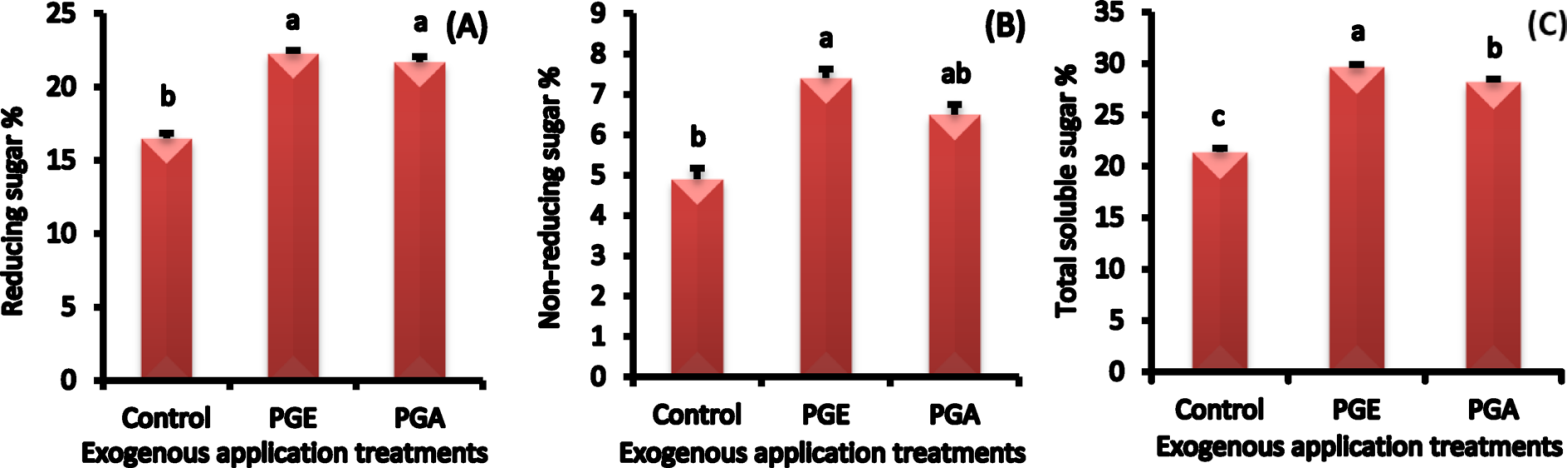
Reducing **(A)**, non-reducing **(B)**, and total soluble sugars **(C)** concentrations in ‘Zaghloul’ date palm fruits as affected by exogenous applications of pollen grain of aqueous **(PGA)** or ethanolic **(PGE)** extract. Values are mean ± standard error (*n* = 3). Letters above the bars indicate significant differences at *p* < 0.05 level following Duncan’s test.

Application of aqueous or ethanolic pollen extract significantly increased the different sugar concentrations of the ‘Zaghloul’ fruit as compared to control (Fig. 4A, B, and C). Fruits treated with ethanolic pollen extract showed the highest concentrations of reducing, non-reducing, and total soluble sugars reaching over control 35, 51, and 39%, respectively.

#### Total carbohydrate and total protein concentrations

**Fig. 5:**
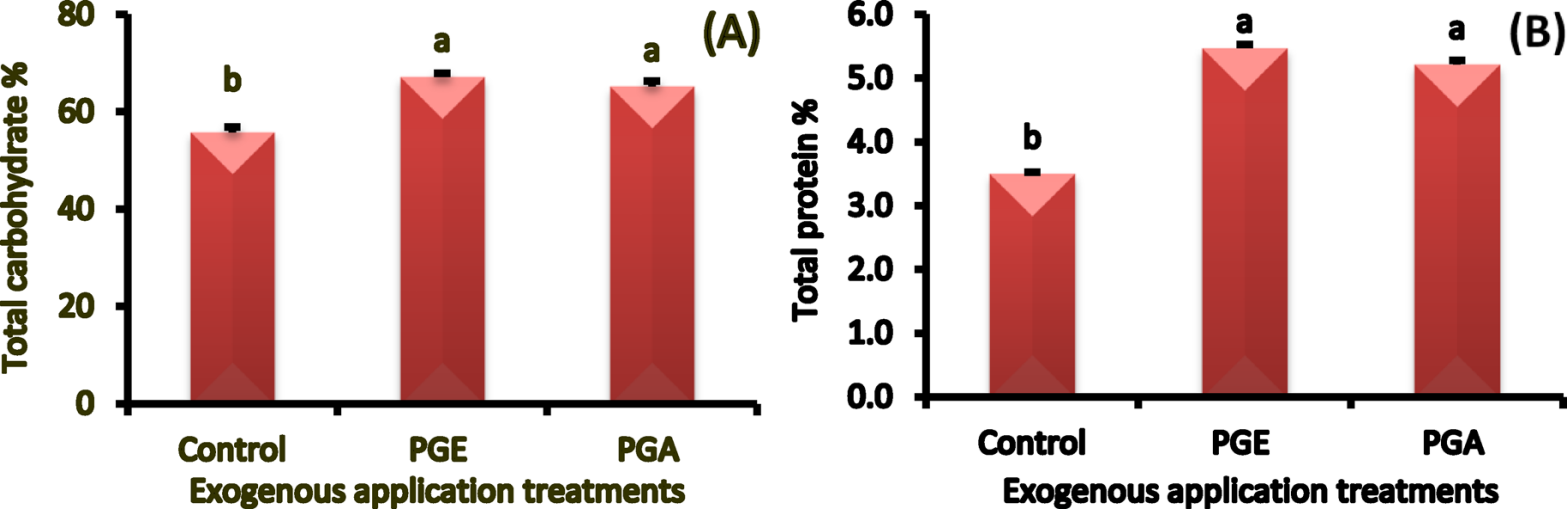
Total carbohydrate **(A)** and total protein **(B)** concentrations in ‘Zaghloul’ date palm fruits as affected by exogenous application of pollen grain of aqueous **(PGA)** or ethanolic **(PGE)** extract. Values are mean ± standard error (*n* = 3). Letters above the bars indicate significant differences at *p* < 0.05 level following Duncan’s test.

Application of exogenous either pollen grains extract i.e., aqueous or ethanolic significantly increased total carbohydrates (17 and 20%, respectively.) and protein (49 and 57%, respectively) concentrations in date fruits as compared to the control treatment (Fig. 5A and B). The date fruits treated with ethanolic pollen extract showed the highest concentration of total carbohydrate concentration as compared to the control fruits.

#### Amino acids concentrations

**Fig. 6:**
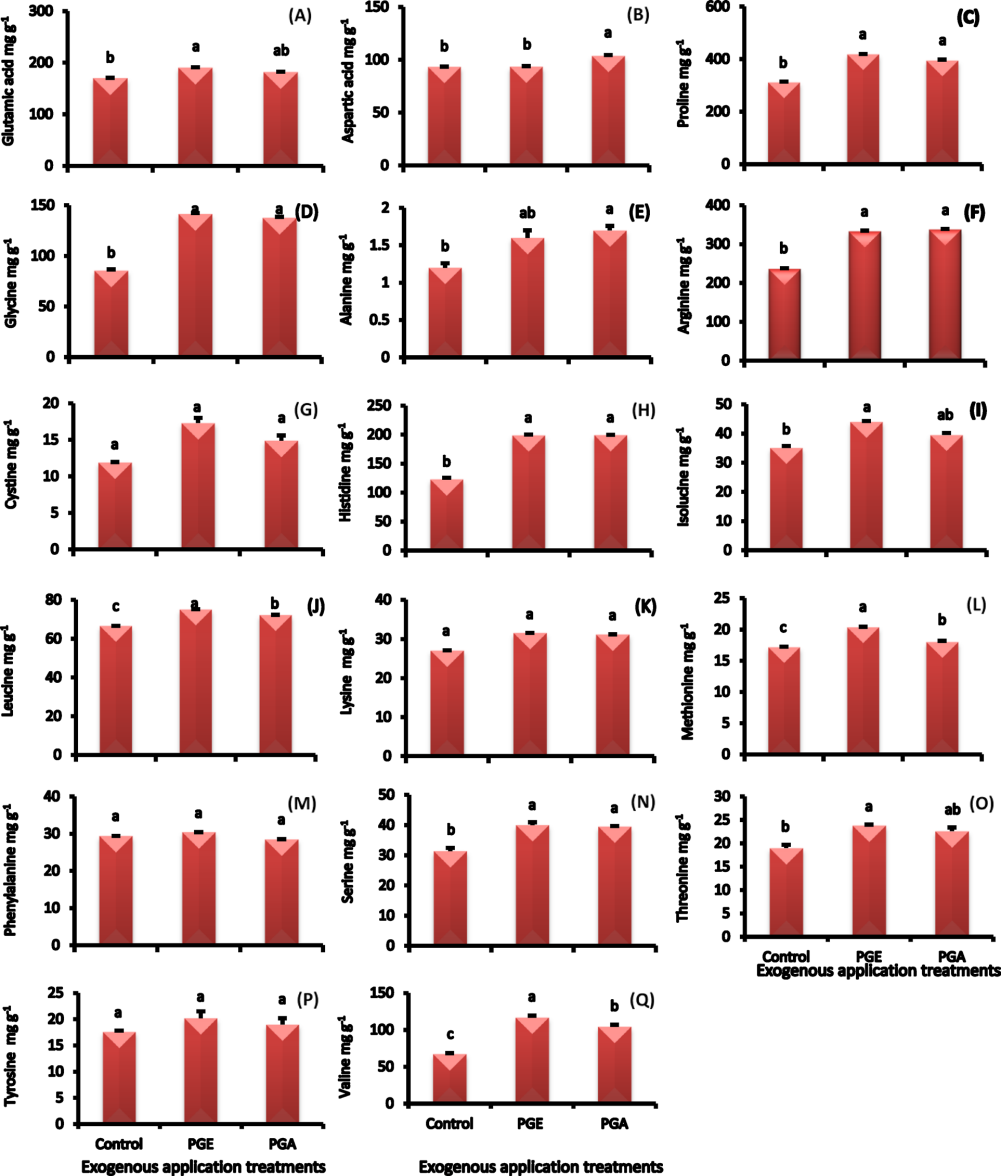
Glutamic acid **(A)**, aspartic acid **(B)**, proline **(C)**, glycine **(D)**, alanine **(E)**, arginine **(F)**, cysteine **(G)**, histidine **(H)**, isoleucine **(I)**, leucine **(J)**, lysine **(K)**, methionine **(L)**, phenylalanine **(M)**, serine **(N)**, threonine **(O)**, tyrosine **(P)**, and valine **(Q)** concentrations in ‘Zaghloul’ date palm fruits as affected by exogenous applications of pollen grain of aqueous **(PGA)** or ethanolic **(PGE)** extract. Values are mean ± standard error (*n* = 3). Letters above the bars indicate significant differences at *p* < 0.05 level following Duncan’s test.

The application of pollen grain extracts significantly increased the concentration of amino acids in date fruits (Fig. 6A-Q). When compared to the untreated control fruits, the date fruits treated with ethanolic pollen grain extract showed a significant increase in amino acid concentration over control reaching for glutamic acid, proline, glycine, cysteine, isoleucine, leucine, lysine, methionine, threonine, and valine by 12, 34, 65, 45, 25, 13, 17, 19, 25 and 74%, respectively. On the other hand, aspartic acid and alanine established highest values with aqueous pollen grain extract reaching over ethanolic pollen grain extract or control by 11 and 42%, respectively. Meanwhile, the aqueous or ethanolic extract recorded similar increase in arginine, histidine and serine concentrations over the control. In addition, there is no change in fruit phenylalanine concentration among treatments and control.

#### Mineral nutrients concentrations

**Fig. 7:**
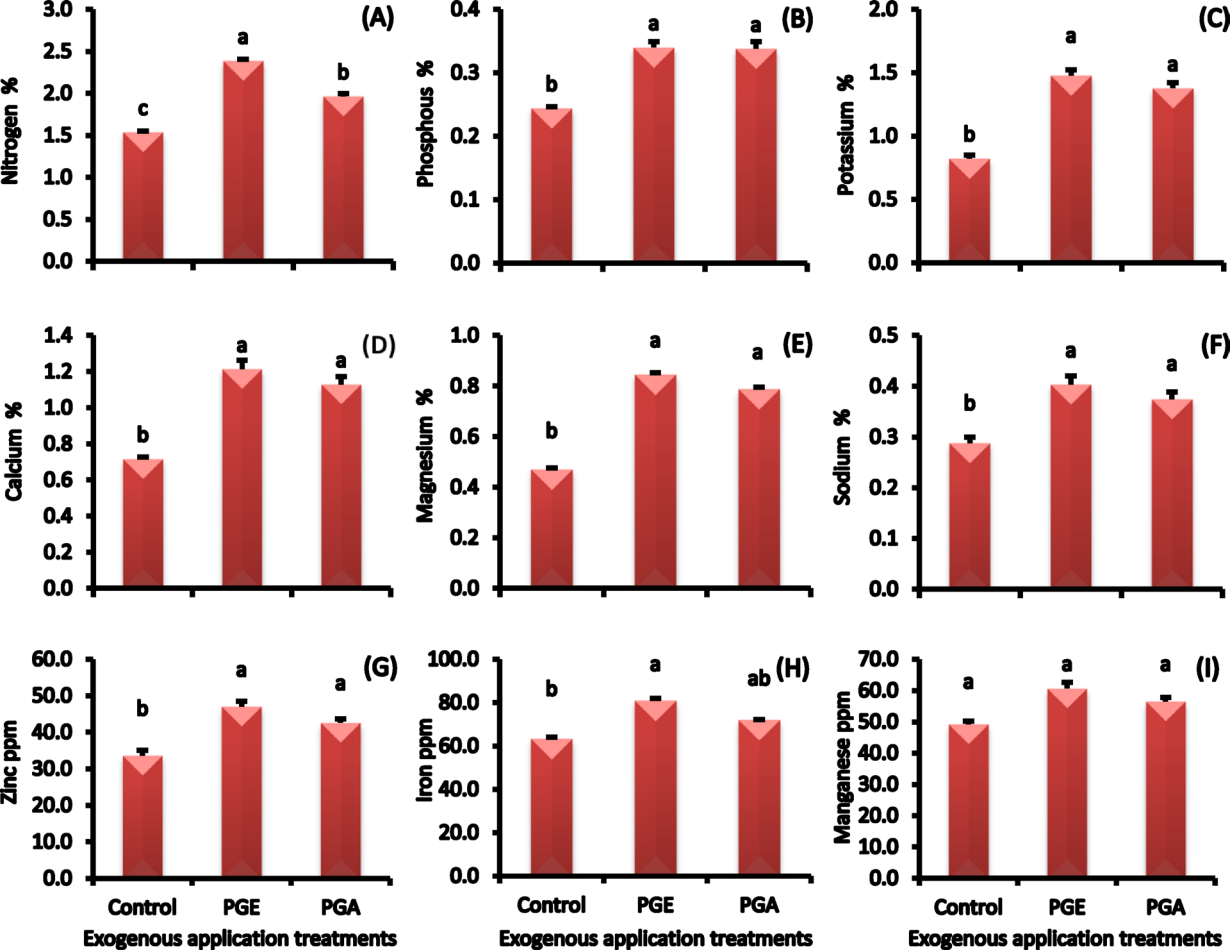
Nitrogen **(A)**, phosphorus **(B)**, potassium **(C)**, calcium **(D)**, magnesium **(E)**, sodium **(F)** concentrations, as well as zinc **(G)**, iron **(H)**, and manganese **(I)** concentrations (ppm) in ‘Zaghloul’ date palm fruits as affected by exogenous applications of pollen grain of aqueous **(PGA)** or ethanolic **(PGE)** extract. Values are mean ± standard error (*n* = 3). Letters above the bars indicate significant differences at *p* < 0.05 level following Duncan’s test.

The application of either pollen grain extract significantly increased the mineral nutrients concentrations in ‘Zaghloul’ fruit as compared to the control treatment (Fig. 7A-I). The date fruits treated with ethanolic pollen extract showed a significant increase in mineral nutrients concentrations over aqueous pollen grain extract or control. The increase in concentrations of nitrogen, phosphorus, potassium, calcium, magnesium, sodium, zinc, iron, and manganese over the control reaching 60, 50, 88, 71, 60, 33, 39, 28, and 23%, respectively.

#### Antioxidant profile

The experimental treatments demonstrated distinct effects on both enzymatic and non-enzymatic antioxidant systems in ‘Zaghloul’ fruits.

##### Non-enzymatic antioxidants

**Fig. 8:**
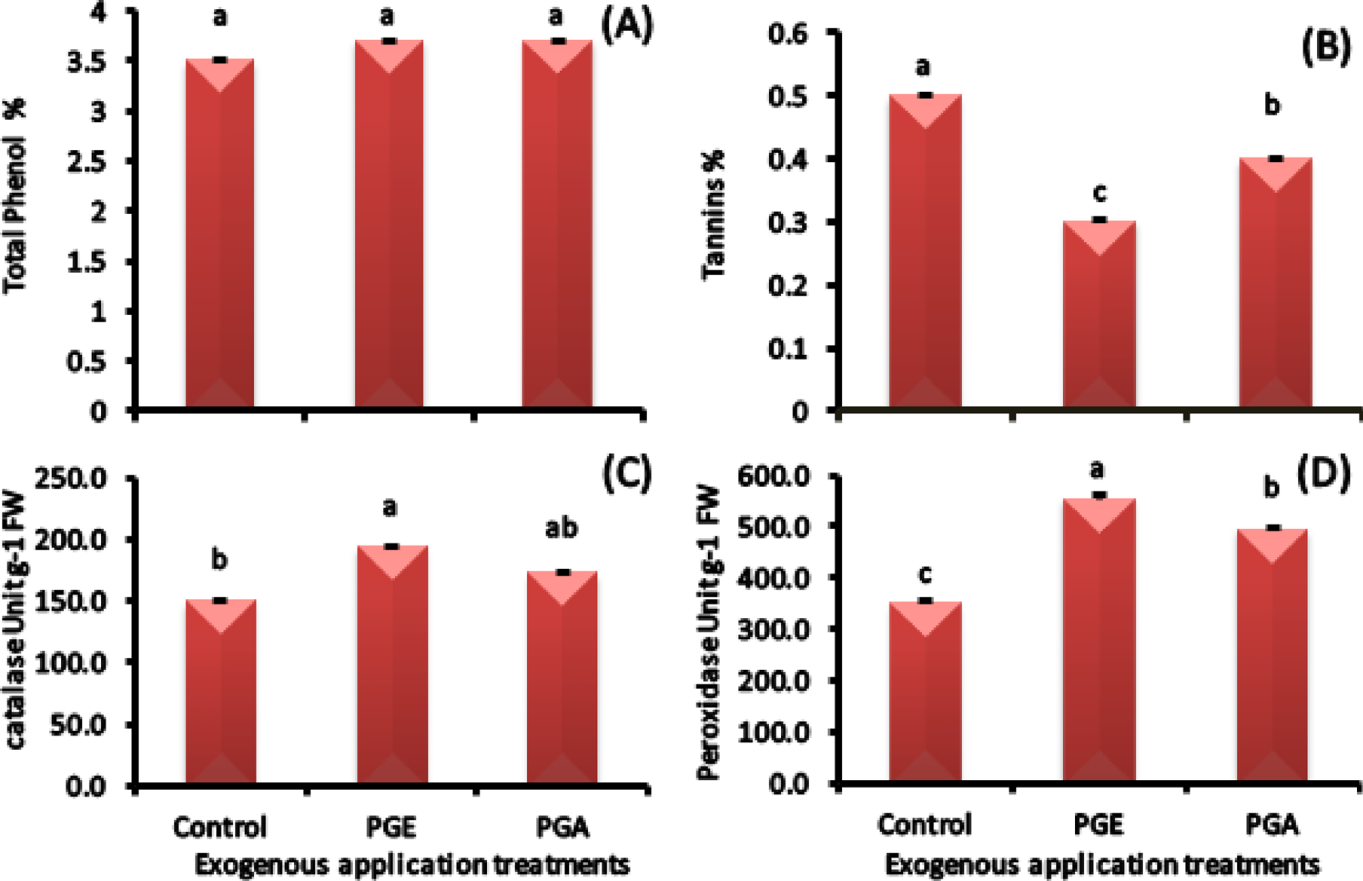
Total phenol **(A)** and tannins **(B)** concentrations as well as catalase **(C)** and peroxidase **(D)** activities in ‘Zaghloul’ date palm fruits as affected by exogenous applications of pollen grain of aqueous **(PGA)** or ethanolic **(PGE)** extract. Values are mean ± standard error (*n* = 3). Letters above the bars indicate significant differences at *p* < 0.05 level following Duncan’s test.

Both extract types elicited a consistent 6% increase in total phenols concentration. In contrast, tannins content exhibited a marked reduction, decreasing by 20% under aqueous extract treatment and more substantially by 40% with ethanolic extract application (Fig. 8A and B).

##### Antioxidant enzymes activities

The ethanolic extract treatment produced a 29% increase in catalase (CAT) activity and a more pronounced 59% enhancement in peroxidase (POD) activity as compared to control. The aqueous extract showed more moderate but still significant increases of 15% for CAT and 41% for POD activity (Fig. 8C and D).

## Discussion

The palm pollen grain extract is rich in protein, mineral nutrients, amino acids, and antioxidants (Table 1). In the current study, we found that application of pollen grain either aqueous or ethanolic extract improved bunch weight of Zaghloul date palm. This might be due to the positive impact of pollen grain extract on improving the physical and chemical properties of fruits as demonstrated in the obtained results which were consistent with the previous studies reporting that many plants extract application promotes fruit growth of different plants^29,30^. In this concern, Mannino et al. (2020)^31^ stated that plant biostimulants materials improved fruit development and quality as well as they are free from chemical contaminants.

Total soluble solids (TSS) represented the organic acids, minerals, fats, proteins, and carbohydrate found in fruits, while titratable acidity represented only the amount of organic acids present in the fruit. The present study showed that spraying aqueous or ethanolic pollen grain extract on date palm fruits can improve fruit flavor through increasing the total soluble solid, and sugar whereas decreasing the titratable acidity of date fruits. Reduced acidity in fruits might be due to enhancing delivery of sugars to fruit tissues, and conversion of organic acids into sugars as mentioned by Soppelsa et al.. (2018)^32^ who found that the higher total soluble solid is due to enhancement of metabolic activities of the fruit, resulting from the synthesis of greater amount of metabolites, glucose, and acids through bio-stimulant treatments. They ascribed that organic acids will turn into sugars upon maturity, which gives an additional fruit taste to the treated fruits. The GC-MS analysis, following the methodologies of Ghanem et al. (2015) and Abdallah et al. (2023)^33,34^, identified key volatile compounds exclusively in the ethanolic extract of date palm pollen (DPP), including ethyl oleate, ethyl-9-hexadecenoate, and 9,12,15-octadecatrienoic acid-ethyl ester, establishing the biochemical basis for observed flavor enhancement. These secondary metabolites, resulting from pollen extract-induced upregulation of fatty acid biosynthesis pathways, which could correlate with sensory panel evaluations. The dominant ethyl oleate, exhibiting a high concentration, particularly contributed to the perceived fruity sweetness, while collectively, these esters explain the flavor score improvement versus controls through direct aroma substances production, and sugar-acid balance modulation.

In the current study, palm pollen extract treatments increased different fruit sugar concentrations. This result is consistent with Sani et al.. (2020) and Subroamaniyan et al.. (2023)^35,36^. Concerning the accumulation of total carbohydrate in fruits, the exogenous pollen extracts showed an increase in carbohydrate concentration in ‘Zaghloul’ fruits. According to Genard et al.. (1991)^37^, glycine retards chlorophyll degradation during storage conditions in isolated chloroplasts, so, glycine in pollen grain extract might be responsible for enhancing photosynthesis in date fruits and their reflection on carbohydrates accumulation.

Our results align with the studies highlighting the role of date palm pollen grains extract in fruit development with El-Shiekh and Umaharan (2013)^38^ who reported similar improvements in ‘Khalas’ dates, linking date palm pollen extract to enhanced sugar accumulation and fruit set. Moreover, the same researchers found that date palm pollen extract and gibberellic acid (GA_3_) synergistically increased fruit size and delayed ripening in ‘Khalas’ dates, supporting our observations from pollen grain extracts efficacy. The superior performance of pollen grain extracts may stem from its rich composition of mineral nutrients that could penetrate into tissues showing an additional mechanism^11^. Mineral, protein, and carbohydrate profiles of date palm pollen grains demonstrate a sophisticated biostimulant capacity that operates through multiple physiological pathways^39^.

Various essential elements available in date palm fruit improve their nutritional value^40^. Our findings showed that the pollen grain extract treatments increased the concentration of mineral nutrients in the ‘Zaghloul’ fruit. This might refer to the influence of pollen extract on the source-sink relationship and the allocation of nutrients to the reproductive organs by the effect of the chemical constituents of the pollen extract. Furthermore, the mineral nutrients (phosphorus, potassium, calcium, magnesium, iron, manganese, and zinc) enhance the nutritional quality of food products^41^. In addition, the significant increase in nutrient uptake and efficiency by biostimulants enhanced plant growth^42^, and the significant increase in potassium (K⁺) concentration enhances sugar transportation, while comprehensive mineral profiling revealed coordinated increase in nitrogen, phosphorus, calcium, magnesium, and micronutrients (zinc, iron, manganese), indicating systemic improvement in nutrient allocation to developing fruits.

The amino acids present in pollen grain extract could maintain higher antioxidant defense system through increasing activities of catalase (CAT) and peroxidase (POD). Enzymatic and non-enzymatic antioxidants in pollen grain extracts are thought to be the mechanisms that enable improvement of Zaghloul date palm production. Our findings are in consistent with those of Taha et al. (2020)^11^ who found an increase in antioxidant enzyme activities in basil plants treated with pollen grain extract that might be due to the roles of the pollen constituents in enhancing nutritional status and fruit growth reflected on the date fruit quality. In addition, palm pollen extract increased the phenolic content in Zaghloul date palm fruits; which might be attributed also to the effect of antioxidants. On the other hand, the enhanced enzymatic activity, coupled with increase in total phenolic content, suggests that the constituents of pollen extract function through multiple complementary pathways directly by reactive oxygen species scavenging via enzymatic (catalase and peroxidase) and non-enzymatic (phenolic compounds) antioxidants; which could improve nutritional status through amino acid-mediated nitrogen metabolism; and potential upregulation of secondary metabolite biosynthesis. The parallel increase in flavor-related metabolites and antioxidant capacity suggested that date palm pollen grain extract treatment might synergistically improve both organoleptic quality and postharvest stability of Zaghloul dates, contributing to extended shelf life and improved stress tolerance. Antioxidant activity was observed in date palm pollen extracts by Abou Zeid et al. (2019)^43^, attributed to their rich phenolic and flavonoid composition. In this regard, presence of various antioxidants, i.e. ascorbic acid, phenols, flavonoids and β-carotene could protect fruit phenolic content from oxidation and thus enhance their concentration in fruit^44^.

The ethanolic extract consistently outperformed the aqueous extract across all measured parameters, showing superior efficacy in improving fruit weight, volume, and pulp percentage, as well as enhancing biochemical markers including total soluble solids, sugar content, protein levels, and amino acid concentrations. This superiority was particularly evident in glutamic acid levels, which showed a 12% greater increase in PGE-treated fruits compared to PGA. This enhanced performance of PGE is likely attributable to its greater extraction efficiency for bioactive compounds^45^, resulting in 2.1-fold higher amino acid content, significantly elevated mineral levels (particularly potassium and calcium), and substantially improved enzymatic activities of catalase and peroxidase compared to the aqueous extract.

Finally, palm pollen grain extract might act as a source of growth-substance materials such as mineral nutrients, amino acids, antioxidants, and proteins (Table 1). Many biostimulants have been extracted from plant tissues and applied to improve both crop yield and quality as well as plant defense systems^46^. In this regard, the influence of pollen grain extract as exogenous application on basil plants productivity and its growth characteristics, has been investigated^11^. Therefore, pollen grain extract could be used as a powerful growth biostimulant as it is considered a sustainable, environment friendly efficient strategy and inexpensive tool to replace using commercially synthesized materials, which might be expensive and unsafe. For the time being, researchers frequently use biostimulant plant extracts to improve crops under normal and stress conditions^47,48^.

## Conclusion

We can conclude from our results that the use of pollen grain extracts seems to be useful in encouraging the synthesis of substantial substances such as sugar and amino acids and stimulate their transfer to the developing fruit, which leads to an increase in the weight of date fruits and its quality. Moreover, palm pollen grain extracts either aqueous or ethanolic, not only improve fruit weight, but also enhanced fruit quality by regulating endogenous sugar, amino acids, mineral nutrients, carbohydrate, and protein fruit content. Therefore, the use of pollen grain extract, especially of ethanolic, as a plant biostimulant could improve the internal physiology of the developing fruits by ensuring that they receive an adequate supply of nutrients and other compounds required for proper growth and development, leading to increased fruit size and quality.

## Data Availability

The authors declare that the raw data supporting the findings of this study are available and can be requested from the corresponding author when needed.
